# Record of a Few Cases of Abdominal Surgery

**Published:** 1897-11

**Authors:** E. Mayhew Michener

**Affiliations:** North Wales, Pa.


### REPORTS OF CASES.

### RECORD OF A FEW CASES OF ABDOMINAL SURGERY.[1]

By E. Mayhew Michener, V.M.D.,
NORTH WALES, PA.

Case I. *Difficult parturition in a sow, with operation.*—Subject, Berkshire sow, reported by owner to have been in labor forty-eight hours; no delivery. On examination found one pig presenting; anterior presentation. After considerable work delivered this one pig. After waiting four hours for more, and finding there was no progress and sow growing weaker, I decided upon Cæsarean section (gastro-hysterotomy). I secured the sow on the left side and scrubbed the right side thoroughly with soap and water; clipped the bristles closely over about a square foot of surface surrounding the line of incision, then washed with a 10 per cent. solution of creolin, and the surface wiped dry with a clean towel. I incised the skin about six inches from a point two inches in front of extreme angle of the ilium downward and forward, in line with the fibres of the small oblique muscle of the abdomen, which was incised for nearly the same distance, however not as far anteriorly and downward as was the incision through the skin. The transverse fascia was then incised to a like extent and the peritoneum lifted up by rat-tooth forceps, and an opening made large enough to admit the finger, which was then used as a guide for the probe-pointed bistoury, which was used to enlarge the opening in the peritoneum enough to admit the hand. The uterus, containing the young, was easily secured, and the part of the horn containing the pig was brought out through the opening and found very near the divergence of the two horns. An incision was made at a point as near as possible to the body of the uterus and the pig extracted by means of a strong tenaculum placed in the snout. Care was taken that the fluids escaping with the fœtus did not touch the wound in the abdomen, nor fall into the abdominal cavity. Two other pigs, which were found to be in the distant horn of the uterus, were removed through the same opening. All were dead. The placentæ were easily removed, and all fluids were pressed out.

[1] Presented for consideration at the semi-annual meeting of the Pennsylvania State Veterinary Medical Association, Franklin, Pa., September, 1897.

The wound in the uterus was washed with warm water containing creolin (one part to twenty), and at once stitched up with interrupted suture of waxed linen thread. Care was taken to invert the edges of the wound so that the peritoneal surface of the uterus came into close contact throughout the length of the incision. In placing the stitches the needle was carried through the peritoneal and muscular coats of the uterus, but *not* through the mucous-membrane coat. The stitches were placed one-half inch apart. The uterus was returned, and the wound in the abdominal wall closed by two rows of stitches; the first, continuous suture of the peritoneum, the second consisted of two stout wires passed through the skin, one at the upper and the other at the lower third of the incision through the skin. Each wire was then wrapped in the figure-eight fashion with a stout linen suture, thus bringing the cut edges closely together. The space between and beyond the wires was sutured firmly with interrupted sutures, a small opening being left at the lowest point of the wound for drainage. The wound was then wet with a 50 per cent. creolin solution, and the animal released and turned into the orchard. It was very much exhausted and somewhat lame behind on the side of the incision. The weather being very warm, the animal was drenched frequently with water from a pump, which she seemed to enjoy very much. Her appetite was very poor for the first three days, then rapidly improved, and in eight days apparently all right. In one month she was rapidly gaining flesh.

CASE II. *Difficult parturition in a sow.*—Chester-white sow; in labor two days, during which time four pigs were delivered by much assistance being given. The sow then seemed to be entirely undisturbed by labor pains for about ten hours, and then began to labor feebly and without any result. Operation decided upon and method described in Case I. followed. Nothing different from Case I., except I found a considerable quantity of fluid in the abdominal cavity, which poured out on incising the peritoneum; on exposure to the air the liquid turned from transparent to almost white, resembling heated albumin, and from very liquid to the consistency of jelly. In removing the pigs, which were four, I found it necessary to make an incision in each side of the uterus. Wounds in uterus and abdominal wall closed as before described. Animal very much depressed, and little appetite for nearly a week, then began to improve slowly, and in three weeks was gaining flesh.

CASE III. *Operative interference in bitch in difficult parturition.*—Fox terrier bitch; in labor fifteen hours, during which time four puppies (two alive and two dead) were born. The fifth pup I was unable to secure, it not coming back within reach. Secured the animal, cleansed the abdomen with soap and water, disinfected with creolin solution; made an incision on the median line from just anterior of the pelvis to near the umbilicus. The uterus was drawn through the wound and found to contain only one pup, which was well back in the body of the uterus. Incised the right horn and extracted the pup alive. Wound in the uterus sutured and wound in the abdomen closed by close interrupted sutures, including both skin and recti muscles. Animal slightly depressed after operation, and the temperature, which was before 102°, rose to 104½° in less than two hours, but fell to 101° four hours later. Bitch drank some milk and raw eggs three hours after operation, and the next day the appetite was good. Pup alive and nursing, but was killed by accident when four days old. This animal made a complete recovery, and showed throughout less disturbance than I have frequently witnessed following ovariotomy.

CASE IV. *Operation and delivery of puppies in difficult parturition.*—French poodle bitch; in labor three days, during which time three pups were delivered. I found the animal very weak with a temperature of 105°; labor pains weak. I succeeded, by hard work, in extracting one more pup badly decomposed. As, from appearances, no more were likely to be secured I informed the owner that I possibly might save the bitch's life by an operation, as a last resort. He consented, and I proceeded as with the fox terrier, and obtained eight more dead and partly decomposed young. The bitch was very weak and threatened collapse after finishing the operation. I placed her in a warm room, and gave small repeated doses of brandy and milk. She seemed to gain slowly until about forty-eight hours after the operation, then failed rapidly and died on the third day. The autopsy showed very slight peritonitis, but marked inflammation of the womb. The incisions in the womb had become fastened quite tightly together with lymph-bands, making an almost perfect closure.

CASE V. *Laceration of intestines after castration in a boar; recovery.*—I wish to describe here an accident which occurred during an operation upon a ridgeling boar. I have frequently performed this operation, almost always with success, on boars of ages varying from a few months to three years. The method used is to secure completely on an inclined plane, the head placed downward. The incision is made as in the operation described on sows. The testicle, or sometimes both, have no certain location, varying from the lumbar region to the internal inguinal ring; generally, however, it is found well down toward the internal ring.

The accident which I wish to describe consisted of a transverse tear in the small intestines, severing the gut quite one-half its circumference. It was caused by using too great force in attempting to return the intestines through the opening in the abdominal wall, through which they had been forced by the violent struggles of the animal.

I informed the owner, who was present, that most likely the accident would prove fatal, but repaired the injury to the gut by turning the torn edges inward and suturing with silk thread and continuous suture, going only through the peritoneal coating of the bowel. Upon inquiry several weeks later, I was not a little surprised and pleased to learn that the pig never showed the least incovenience from the combined operations of removing the testicle from the abdomen, another from the scrotum, and suturing the bowel.

